# Interferon-λ Contributes to Innate Immunity of Mice against Influenza A Virus but Not against Hepatotropic Viruses

**DOI:** 10.1371/journal.ppat.1000151

**Published:** 2008-09-12

**Authors:** Markus Mordstein, Georg Kochs, Laure Dumoutier, Jean-Christophe Renauld, Søren R. Paludan, Kevin Klucher, Peter Staeheli

**Affiliations:** 1 Department of Virology, University of Freiburg, Freiburg, Germany; 2 Ludwig Institute for Cancer Research, University of Louvain, Brussels, Belgium; 3 Department of Medical Microbiology and Immunology, University of Aarhus, Aarhus, Denmark; 4 ZymoGenetics, Inc., Seattle, Washington, United States of America; Mount Sinai School of Medicine, United States of America

## Abstract

Virus-infected cells secrete a broad range of interferon (IFN) subtypes which in turn trigger the synthesis of antiviral factors that confer host resistance. IFN-α, IFN-β and other type I IFNs signal through a common universally expressed cell surface receptor, whereas IFN-λ uses a distinct receptor complex for signaling that is not present on all cell types. Since type I IFN receptor-deficient mice (*IFNAR1^0/0^*) exhibit greatly increased susceptibility to various viral diseases, it remained unclear to which degree IFN-λ might contribute to innate immunity. To address this issue we performed influenza A virus infections of mice which carry functional alleles of the influenza virus resistance gene *Mx1* and which, therefore, develop a more complete innate immune response to influenza viruses than standard laboratory mice. We demonstrate that intranasal administration of IFN-λ readily induced the antiviral factor Mx1 in mouse lungs and efficiently protected *IFNAR1^0/0^* mice from lethal influenza virus infection. By contrast, intraperitoneal application of IFN-λ failed to induce Mx1 in the liver of *IFNAR1^0/0^* mice and did not protect against hepatotropic virus infections. Mice lacking functional IFN-λ receptors were only slightly more susceptible to influenza virus than wild-type mice. However, mice lacking functional receptors for both IFN-α/β and IFN-λ were hypersensitive and even failed to restrict usually non-pathogenic influenza virus mutants lacking the IFN-antagonistic factor NS1. Interestingly, the double-knockout mice were not more susceptible against hepatotropic viruses than *IFNAR1^0/0^* mice. From these results we conclude that IFN-λ contributes to inborn resistance against viral pathogens infecting the lung but not the liver.

## Author Summary

The contribution of IFN-λ to innate immunity against virus-induced diseases has remained unclear to date as appropriate mouse models were not available. We now present evidence that IFN-λ is involved in the antiviral defense. Mice lacking functional IFN-λ receptors were only slightly more susceptible to influenza virus than wild-type mice, but intranasal administration of IFN-λ efficiently protected IFN-α/β receptor-deficient mice from lethal influenza virus infection and induced the antiviral factor Mx1 in lungs. Mice lacking functional receptors for both IFN-α/β and IFN-λ were hypersensitive and failed to restrict even usually non-pathogenic influenza virus mutants lacking the IFN-antagonistic factor NS1. By contrast, intraperitoneal application of IFN-λ failed to induce Mx1 in the liver of mice and did not protect against hepatotropic viruses. Furthermore, double-knockout mice were not more susceptible against hepatotropic viruses than IFN-α/β receptor-deficient mice, indicating that IFN-λ contributes to resistance against viral pathogens infecting the lung but not the liver.

## Introduction

Viral infection of vertebrate cells triggers innate immune responses, which result in rapid synthesis of IFN and other pro-inflammatory cytokines [1]–[4]. Virus-induced IFN represents a complex mixture of IFN subtypes which act on target cells by engaging two distinct cell surface receptors [5]. All members of the type I IFN family which, in the mouse, includes 14 different IFN-α subtypes, IFN-β, IFN-κ, IFN-ε and limitin, use the same heterodimeric IFN-α/β receptor complex (IFNAR1/2) for signaling [6]. By contrast, signaling by type III IFN family members (in the mouse IFN-λ2 and IFN-λ3) occurs through the heterodimeric interleukin-28 receptor α/interleukin-10 receptor β (IL-28Rα/IL-10Rβ) complex [7],[8]. Although activating distinct receptor systems, IFN-λ and type I IFNs trigger strikingly similar responses in target cells which mostly result from phosphorylation-induced activation of transcription factors STAT-1 and STAT-2 [9],[10]. The IFNAR1/2 complex is present on most if not all nucleated cells, whereas expression of the IL-28Rα subunit seems to be cell type-restricted [11],[12]. Consequently, antiviral protection by type I IFN is observed in most cell types, whereas antiviral protection mediated by IFN-λ is restricted to cells that express functional IL-28R complexes. The spectrum of cell types that respond to IFN-λ *in vivo* is poorly defined. Recent experiments suggested that epithelial cells are the main targets of IFN-λ in the mouse [13].

Information on the contribution of IFN-λ to virus resistance at the level of the whole organism is very limited as mice lacking functional IFN-λ receptors (*IL28Rα^0/0^*) were generated only recently [14]. Unlike knockout mice lacking functional type I IFN receptors (*IFNAR1^0/0^*) that are highly susceptible to a broad spectrum of different viruses [15], *IL28Rα^0/0^* and wild-type mice did not differ significantly in resistance to a large panel of pathogenic viruses [14]. The only observed difference between wild-type and *IL28Rα^0/0^* mice was that treatment of knockout mice with toll-like receptor (TLR) 3 and TLR9 agonists failed to induce resistance to vaginal infection with herpes simplex virus type 2 [14].

Here we used *Mx1^+/+^* mice to investigate the relative contributions of IFN-λ and type I IFN in immunity toward influenza A virus. *Mx1^+/+^* mice differ from standard mouse strains in being fully IFN-competent. They carry functional alleles of the influenza virus resistance gene *Mx1*, which is defective in standard laboratory mice [16]. Consequently, in *Mx1^+/+^* mice, virus-induced IFN activates the *Mx1* gene in addition to other antiviral genes, leading to a more complete innate immune response and more robust resistance to influenza and influenza-like viruses [17],[18]. The *Mx1^+/+^* mouse model system has the power to reveal even subtle defects in antiviral immunity against orthomyxoviruses. It has recently been used to uncover the beneficial effect of IFN-β in influenza virus defense [19]. It was further used to demonstrate that IFN-α might be used to prevent disease induced by highly lethal human H5N1 influenza viruses [17]. Using this experimental system we now demonstrate that IFN-λ contributes to innate immunity against influenza virus but not against two different hepatotropic viruses. These differences in virus susceptibility correlated with the differing ability of virus-induced IFN-λ to activate the *Mx1* gene in lung and liver of *IFNAR1^0/0^* mice.

## Results

### IFN-λ is induced in virus-infected lung and liver of *IFNAR1^0/0^* mice

**Figure 1:**
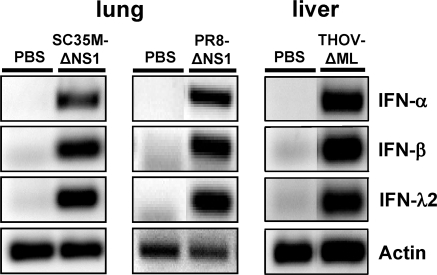
Induction of IFN-λ2 genes in virus-infected lung and liver of *IFNAR1^0/0^* mice. Animals were either infected by the intranasal route with 10^6^ pfu of influenza A virus strain SC35M-ΔNS1 or PR8-ΔNS1, or else by the intraperitoneal route with 10 pfu of hepatotropic THOV-ΔML. Animals treated with plain buffer served as negative controls. At 17 hours post infection, the influenza virus-infected mice were killed and the lungs were removed for analysis. The liver of the THOV-infected mouse was harvested when the animal was severely diseased at 72 hours post infection. RNA samples from the organs were reverse transcribed and analyzed by PCR for transcripts of the indicated genes.

Since virus-induced activation of IFN genes requires positive feedback through the IFN-α/β receptor in certain cell types [20], we first determined whether the major IFN subtypes are induced in lung and liver of *IFNAR1^0/0^* mice after infection with viruses that strongly activate the innate immune system. As can be seen in Figure 1, we observed strong transcriptional activation of genes for the IFN-α family, IFN-β and IFN-λ2 in the lung of mice infected intranasally with the influenza A virus mutants SC35M-ΔNS1 and PR8-ΔNS1 that are known to induce large amounts of type I IFN [21],[22]. Similarly, strong transcriptional activation of IFN-α, IFN-β and IFN-λ genes was observed in the liver of *IFNAR1^0/0^* mice infected with a mutant of hepatotropic Thogotovirus (THOV) that lacks the IFN-antagonistic factor ML (THOV-ΔML) [23].

### Exogenous IFN-λ protects *IFNAR1^0/0^* mice against intranasal challenge with influenza A virus but not against intraperitoneal challenge with Thogotovirus

**Figure 2:**
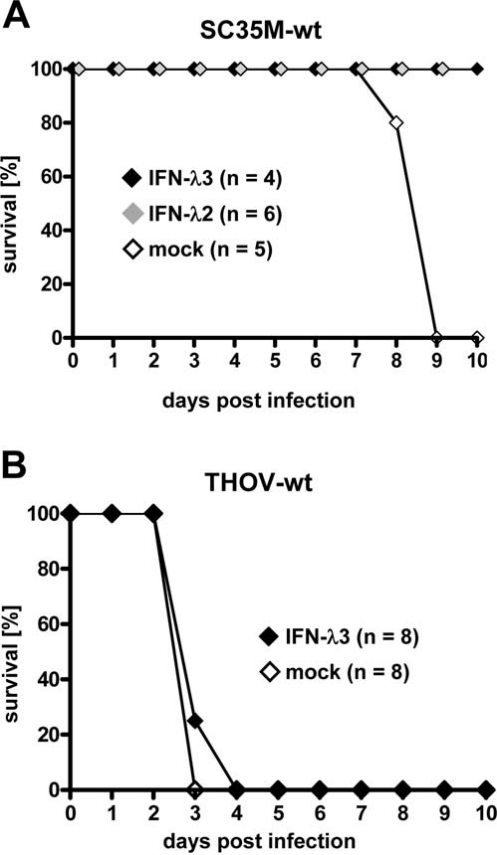
Exogenous IFN-λ protects *IFNAR1^0/0^* mice against intranasal challenge with influenza A virus but not against intraperitoneal challenge with THOV. (A) Survival of mice intranasally treated for 10 hours with a mock preparation or 7,500 units of either IFN-λ2 or IFN-λ3 before challenge with 100 pfu (∼20 LD_50_) of influenza A virus strain SC35M. (B) Survival of mice intraperitoneally treated for 10 hours with a mock preparation or 15,000 units of IFN-λ3 before infection with 100 pfu (∼20 LD_50_) of THOV.

In a first experiment, *IFNAR1^0/0^* mice were treated with exogenous IFN-λ by the intranasal route to determine whether this cytokine might contribute to protection from pneumonia induced by pathogenic influenza viruses. Groups of *IFNAR1^0/0^* mice were treated with 7,500 units of either recombinant IFN-λ2 or IFN-λ3. Control animals received corresponding volumes of a mock preparation. Ten hours later, the animals were infected with 100 plaque-forming units (pfu) (∼20 LD_50_) of mouse-adapted H7N7 influenza A virus strain SC35M [22]. The control animals quickly lost weight and had to be killed between days 7 and 9 post infection due to clinical signs of pneumonia, whereas all animals treated with either IFN-λ2 or IFN-λ3 remained healthy (Fig. 2A). Since standard *IFNAR1^0/0^* mice lacking functional *Mx1* alleles developed severe disease under identical experimental conditions in spite of treatment with IFN-λ3 (data not shown), we concluded that the protective effect of IFN-λ that we observed in our *Mx1^+/+^* mice was mainly mediated by the IFN-induced resistance factor Mx1.

In a second experiment, 15,000 units of IFN-λ3 were applied by the intraperitoneal route to *IFNAR1^0/0^* mice carrying functional Mx1 alleles. Ten hours later the animals were challenged with 100 pfu (∼20 LD_50_) of THOV. Animals treated with IFN-λ3 as well as control animals treated with a mock preparation developed severe disease between 48 and 96 hours post infection (Fig. 2B). Thus, IFN-λ exhibited effective antiviral activity in the lung, but seemed to be inactive in the liver.

### IFN-λ activates *Mx1* gene expression in the lung but not liver of *IFNAR1^0/0^* mice

**Figure 3:**
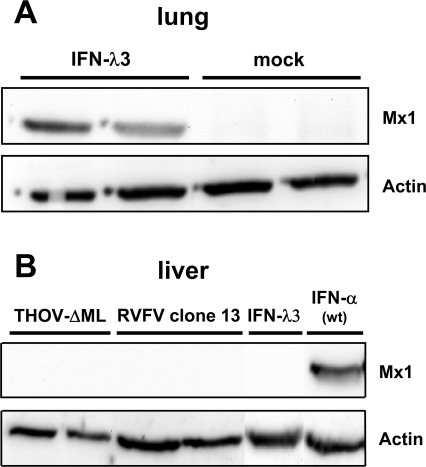
IFN-λ activates *Mx1* gene expression in lung but not liver of IFNAR1^0/0^ mice. (A) Western blot analysis of Mx1 protein levels in lungs of mice at 20 hours post intranasal application of 3,500 units of IFN-λ3. Animals treated with a mock preparation served as control. Two animals of each group are shown. (B) Mx1 protein levels in the liver of *IFNAR1^0/0^* mice at 20 hours post intraperitoneal application of 15,000 units of IFN-λ3 or terminally ill at 72 hours post infection with hepatotropic THOV-ΔML or RVFV clone 13. Two animals for each group are shown. Liver extract from a wild-type mouse killed at 20 hours post intraperitoneal treatment with 100,000 units of human IFN-αB/D served as positive control.

Like type I IFN, IFN-λ exhibits antiviral activity by binding to a specific cell receptor complex that can activate latent STAT transcription factors [24]. After activation, the STAT proteins move to the nucleus where they activate transcription of a large number of IFN-responsive genes, including *Mx1*. To determine whether exogenous IFN-λ activates IFN-responsive genes in our *IFNAR1^0/0^* mice, we harvested lung and liver at 20 hours post onset of treatment with IFN-λ3 and analyzed the tissue homogenates for Mx1 protein by western blotting. Easily detectable levels of Mx1 were present in lungs of mice that were treated intranasally with 3,500 units of IFN-λ3 (Fig. 3A). The lungs of mock-treated mice did not contain detectable levels of Mx1.

Mx1 protein could not be detected in the liver of *IFNAR1^0/0^* mice treated with 15,000 units of IFN-λ3 by the intraperitoneal route (Fig. 3B). If, as a control, a cross-reactive variant of human IFN-α was injected by the same route into wild-type mice, Mx1 was prominently induced in the liver (Fig. 3B). To distinguish between the possibility that liver cells lack functional receptors for IFN-λ and the possibility that the recombinant IFN-λ failed to reach the liver under our experimental conditions, we analyzed the Mx1 protein levels in *IFNAR1^0/0^* mice infected with THOV-ΔML which strongly induces IFN-λ in the liver (Fig. 1). The liver of mice with severe THOV-induced disease contained no detectable amounts of Mx1 protein (Fig. 3B). Similarly, no Mx1 protein could be detected in the liver of terminally ill *IFNAR1^0/0^* mice infected with Rift Valley fever virus clone 13 (Fig. 3B), another hepatotropic virus with strong IFN-inducing activity [25]. Thus, differential IFN-λ receptor expression in lung and liver seemed to explain why exogenously applied IFN-λ protected *IFNAR1^0/0^* mice from virus-induced disease of the lung but not the liver.

### Slightly reduced resistance to influenza virus of mice lacking functional IFN-λ receptors

**Figure 4:**
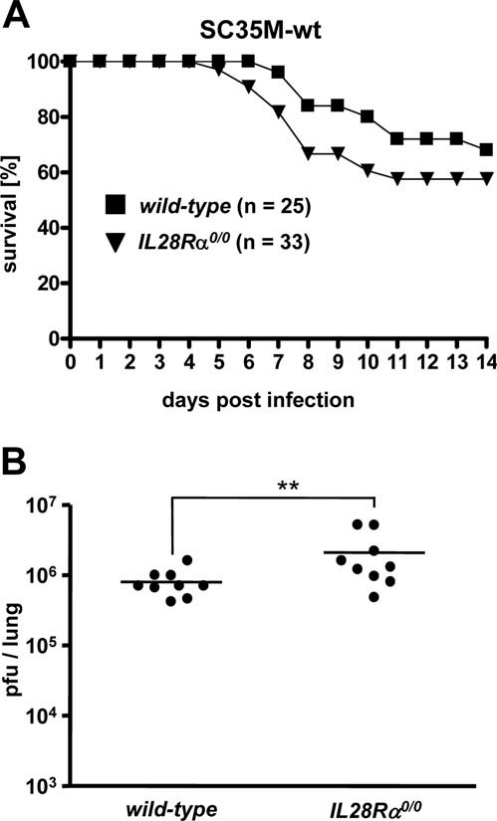
Mice lacking functional receptors for IFN-λ show slightly reduced resistance to influenza A virus. Wild-type and *IL28Rα^0/0^* mice were infected by the intranasal route with 5×10^4^ pfu of SC35M. (A) Survival and (B) virus titers in lungs at 72 hours post infection were recorded. Combined data of several independent experiments are shown. (**: p<0.01).

To directly assess the contribution of IFN-λ to the protection from influenza virus-induced lung disease, we generated *IL28Rα^0/0^* mice carrying functional Mx1 alleles by crossbreeding of appropriate mouse strains and compared the fate of wild-type and *IL28Rα^0/0^* mice after challenge with 5×10^4^ plaque-forming units (pfu) of SC35M. Survival of *IL28Rα^0/0^* mice was slightly reduced compared to wild-type mice (Fig. 4A), but the difference was not statistically significant. Viral titers in lungs of *IL28Rα^0/0^* mice were slightly but significantly higher at 72 h post infection than in lungs of wild-type mice (Fig. 4B).

### Strongly reduced resistance to influenza virus but not to hepatotropic viruses of *IFNAR1^0/0^* mice lacking functional receptors for IFN-λ

**Figure 5:**
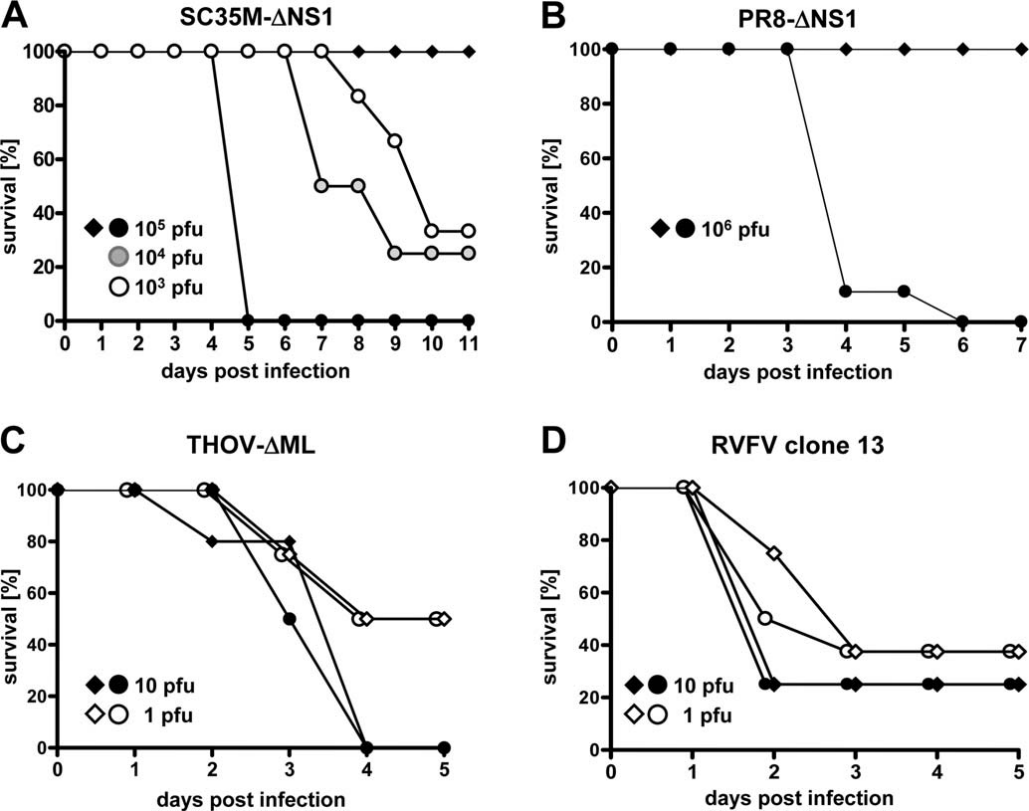
Mice lacking functional receptors for both IFN-α/β and IFN-λ exhibit enhanced susceptibility toward highly attenuated influenza A viruses but not toward two different attenuated hepatotropic viruses. Survival of *IFNAR1^0/0^* (diamonds) and *IFNAR1^0/0^IL28Rα^0/0^* double knockout mice (circles) after (A) intranasal infection with the indicated doses of SC35M-ΔNS1, (B) intranasal infection with 10^6^ pfu of PR8-ΔNS1, (C) intraperitoneal infection with the indicated doses of THOV-ΔML, and (D) intraperitoneal infection with the indicated doses of RVFV clone 13. Groups consisted of four to nine animals.

To determine the relative contributions of IFN-α/β and IFN-λ in antiviral defense we generated *Mx1^+/+^* mice that lack functional receptors for both of these two classes of IFN (*IFNAR1^0/0^IL28Rα^0/0^*) and compared them to mice that lack receptors for IFN-α/β only. We previously demonstrated that *IFNAR1^0/0^* mice with intact *Mx1* alleles are highly susceptible to challenge infections with wild-type SC35M [19]. However, intranasal infection with 10^5^ pfu of SC35M-ΔNS1 did not induce disease in *IFNAR1^0/0^* mice (Fig. 5A). Similarly, all wild-type and *IL28Rα^0/0^* mice remained healthy when challenged with up to 10^6^ pfu of SC35M-ΔNS1 (data not shown). In marked contrast, all *IFNAR1^0/0^IL28Rα^0/0^* double-knockout mice infected with 10^5^ pfu of SC35M-ΔNS1 developed severe disease and had to be killed around day 5 post infection (Fig. 5A). Additional experiments in which we used lower doses of challenge virus demonstrated that the LD_50_ of SC35M-ΔNS1 in *IFNAR1^0/0^IL28Rα^0/0^* double-knockout mice was approximately 10^3^ pfu (Fig. 5A). A similar picture emerged when the mice were challenged with a NS1-deficient variant of the H1N1 human influenza A virus strain PR8 (PR8-ΔNS1). At a dose of 10^6^ pfu, all infected *IFNAR1^0/0^IL28Rα^0/0^* double-knockout mice developed severe pneumonia within 4–6 days post infection, whereas all *IFNAR1^0/0^* single-knockout mice remained healthy (Fig. 5B). Importantly, our single- and double-knockout mice did not differ in susceptibility to infection with the two hepatotropic viruses THOV-ΔML (Fig. 5C) and RVFV clone 13 (Fig. 5D), strongly supporting the above-formulated conclusion that IFN-λ is not active in the liver of *IFNAR1^0/0^* mice.

### High virus load in lungs of mice correlates with low Mx1 protein levels

**Figure 6:**
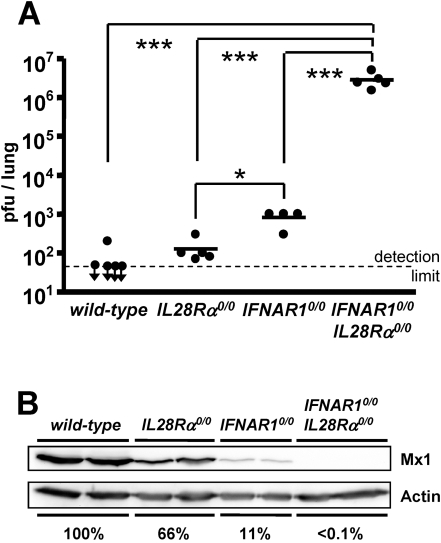
Inverse correlation of Mx1 protein levels and viral load in lungs of mice lacking functional receptors for IFN-α/β, IFN-λ or both. Groups of mice were infected with 10^5^ pfu of SC35M-ΔNS1 and either killed at (A) 48 hours post infection to determine viral titers in the lung or at (B) 20 hours post infection to determine Mx1 protein levels by western blotting. Two animals of each group are shown. Actin-normalized Mx1 signal intensities are indicated. The calculated value of the wild-type mice was set to 100%. (*: p<0.05), ***: p<0.001).

Virus replication in lungs of wild-type and mutant mice was assessed at 48 hours post infection with 10^5^ pfu of SC35M-ΔNS1. Virus titers in lungs of wild-type mice were below the detection limit in four of five animals, and they were only slightly above the detection limit in lungs of *IL28Rα^0/0^* mice at 48 h post infection (Fig. 6A). Remarkably, SC35M-ΔNS1 did not grow much better in lungs of *IFNAR1^0/0^* mice, whereas it replicated to very high titers in lungs of *IFNAR1^0/0^IL28Rα^0/0^* double-knockout mice (Fig. 6A). At 20 hours post infection with SC35M-ΔNS1 the Mx1 protein levels in lungs of *IL28Rα^0/0^* mice were about 2-fold lower than in the wild-type animals (Fig. 6B). Lungs of infected *IFNAR1^0/0^* mice contained about 10-fold lower levels of Mx1 protein than wild-type mice, whereas Mx1 levels were below the detection limit in *IFNAR1^0/0^IL28R*α*^0/0^* double-knockout mice (Fig. 6B). Thus, after infection with SC35M-ΔNS1, the extent of *Mx1* gene expression in lungs of mice with defective receptors for IFN-α/β, IFN-λ or both correlated inversely with virus titers.

## Discussion

The intracellular signaling pathways activated by IFN-λ and IFN-α/β are quite similar [9],[10], suggesting that both IFN types are contributing to virus resistance. Surprisingly, however, mice lacking functional receptors for IFN-λ did not differ from wild-type mice when challenged with a panel of different pathogenic viruses [14]. A mild deficiency of IFN-λ-deficient mice became only apparent in an experimental setting in which resistance to herpes simplex virus type 2 was induced by treating the animals with TLR3 or TLR9 agonists [14]. This phenotype is in marked contrast to that of mice lacking functional receptors for IFN-α/β which are highly susceptible to many viruses [15].

We reasoned that the different phenotypes of the knockout mice might be explained by the different expression patterns of the receptors for IFN-α/β and IFN-λ in the organism. Receptors for IFN-α/β are rather uniformly expressed on most if not all nucleated cells [26], whereas receptors for IFN-λ are preferentially expressed on epithelial cells [13]. If our reasoning was correct, one would predict that the protective effect of IFN-λ should be restricted to organs with a high percentage of cells expressing the IFN-λ receptor and that the protective effect of IFN-λ in these organs might be most obvious when the IFN-α/β system is defective. In this report we provide evidence that strongly supports this view.

We observed that intranasal application of IFN-λ protected the mice from lethal challenge with influenza A virus, whereas systemic application of IFN-λ failed to mediate protection from disease induced by a hepatotropic virus (Fig. 2). It should be noted that the mice employed here lacked functional IFN-α/β receptors, excluding the possibility that the protective effect in the lung was indirect and resulted from IFN-α/β that might have been induced by undefined contaminating substances in our IFN-λ preparations. Protection against influenza virus correlated with the presence of the IFN-induced Mx1 protein in the lung tissue (Fig. 3), suggesting that lung epithelial cells carry functional IFN-λ receptors. By contrast, no Mx1 protein was found in liver tissue of mice treated with IFN-λ (Fig. 3). The liver tissue also failed to respond to IFN-λ synthesized in the virus-infected liver (Fig. 1), suggesting that mouse liver cells do not express functional receptors for IFN-λ. This latter conclusion is in agreement with results from recent quantitative RT-PCR analyses which showed that the alpha chain of the IFN-λ receptor (IL28R-α) is expressed only at very low levels in liver of mice [13]. However, our results appear to be in conflict with a previous report in which IFN-λ was successfully used to inhibit hepatitis B virus replication in a murine hepatocyte cell line expressing the viral genome as a transgene [27]. However, these authors observed no induction of IFN-responsive genes in the liver of mice treated with large amounts of IFN-λ, and they observed no inhibition of hepatitis B virus replication *in vivo* [27]. In this respect, hepatocyte cell lines may not mirror the normal behavior of hepatocytes in intact liver tissue.

Since the virus challenge studies in a former report [14] were carried out with IFN-λ receptor knockout mice lacking the IFN-induced influenza virus resistance factor Mx1, it remained possible that the beneficial effect of IFN-λ against influenza virus had previously been underestimated. Yet, our new experiments with Mx1-positive mice revealed that the lack of IFN-λ system has indeed a much less drastic effect on virus resistance than the lack of the IFN-α/β system. The protective role of IFN-λ became only apparent in Mx1-positive mice that lack a functional IFN-α/β system, and it was most prominent if influenza virus mutants with high IFN-inducing potential were used (Fig. 5). It is well known that highly pathogenic influenza viruses are not controlled well by the IFN system because the virus-encoded NS1 protein counteracts efficient activation of IFN genes in infected cells [21]. NS1-deficient influenza viruses which are very potent IFN inducers are highly attenuated in wild-type mice but remain virulent in mice lacking STAT-1 [21], a transcription factor centrally placed in the signaling pathways of all IFN types [28]. We found that mutants of the influenza virus strains SC35M and PR8 lacking NS1 were completely non-virulent in IFN-α/β receptor-deficient mice and failed to replicate efficiently in the lung of such mice (Figs 5A and 5B), which should not be the case if IFN-α/β was the only IFN subtype that confers resistance to influenza viruses. Our observation that double knockout mice lacking functional receptors for IFN-α/β and IFN-λ are highly susceptible to the NS1-deficient influenza virus mutants clearly demonstrates that IFN-λ provides the residual protection in IFN-α/β receptor-deficient mice.

Some important conclusions can be drawn from our data regarding the role of different IFN types in antiviral immunity. First, the virus defense strategy of the lung is not exclusively based on the IFN-α/β system. Our data clearly demonstrate that the IFN-λ system also contributes to innate immunity against influenza A virus. The second important conclusion from our study is that the IFN-α/β system is dominant over the IFN-λ system. IFN-λ thus appears to be part of a secondary defense system which can fill gaps left by the IFN-α/β system. Future studies will help to distinguish between the possibility that IFN-λ is predominantly active against influenza viruses and the possibility that IFN-λ plays a broader role in the lung and improves innate immunity against other pathogenic viruses that infect the respiratory tract. Evidence in favor of the second possibility includes the observation that IFN-λ also restricted vaccinia virus replication in the lung of mice [29]. We further noted with interest that, reminiscent to the situation with NS1-deficient influenza virus, IFN-α/β receptor-deficient mice are able to restrict the growth of human respiratory syncytial virus in the lung far better than STAT-1-deficient mice [30]. This observation suggests that IFN-λ might also help controlling respiratory syncytial virus. Since receptors for IFN-λ are expressed on epithelial cells of many different organs including lung, stomach and intestine [13], it is conceivable that the physiological role of this cytokine is to protect the host from viral infections via mucosal membranes at many different body sites. An important issue to be addressed in the future is whether IFN-λ might serve a similar role in humans.

## Materials and Methods

### Mice

All animals used were of C57BL/6 genetic background. Congenic B6.A2G-*Mx1* mice [31] carrying intact *Mx1* alleles and B6.A2G-*Mx1*-*IFNAR1^0/0^* mice lacking functional type I IFN receptors [19] were bred locally. C57BL/6 mice lacking functional type III IFN receptors (*IL28Rα^0/0^*) [14] were crossed with B6.A2G-*Mx1* and B6.A2G-*Mx1*-*IFNAR1^0/0^* mice to produce strains with intact *Mx1* alleles and defective alleles for *IL28Rα* only, or *IL28Rα* and *IFNAR1* in combination. Six- to eight-week-old animals were used for all infection experiments, which were performed in accordance with the guidelines of the local animal care committee. Animals were euthanized if severe symptoms developed or body weight loss approached 30% of the initial value.

### Viruses

We used wild-type influenza A virus strains SC35M (H7N7) and A/PR/8/34 (H1N1) as well as mutants SC35M-ΔNS1 [22] and PR8-ΔNS1 [21] lacking the IFN-antagonistic factor NS1. We further used wild-type Thogotovirus (THOV) or mutant THOV-ΔML lacking the IFN-antagonistic factor ML [23], and the attenuated “clone 13” strain of Rift Valley fever virus (RVFV) lacking functional IFN-antagonistic factor NSs [25]. All these viruses are classified as BSL2 pathogens in Germany.

### Virus infections

Animals were anesthetized by intraperitoneal injection of a mixture of ketamine (100 µg per gram body weight) and xylazine (5 µg per gram body weight) before intranasal infection with the indicated doses of the various influenza A viruses in 50 µl PBS containing 0.3% BSA. For THOV and RVFV infections, 100 µl-samples of diluted virus stocks were applied intraperitoneally without anaesthesia.

### Cytokines

IFN-λ2 and IFN-λ3 were produced by transient transfection of 293T cells with appropriate expression plasmids [9]. The biological activity of IFN-λ2 and IFN-λ3 was determined as previously described [32]. Hybrid human IFN-αB/D that is highly active on mouse cells was used as positive control [17],[33].

### IFN treatment of mice

Samples containing the indicated amounts of IFN-λ2 or IFN-λ3 were either applied intranasally (50 µl) to anesthetized animals or injected intraperitoneally (200–300 µl) without anaesthesia.

### Titration of virus in lungs

Lung homogenates were prepared by grinding the tissue using a mortar and sterile quartz sand. Homogenates were suspended in 1 ml of PBS, and tissue debris was removed by low speed centrifugation. Virus titers in supernatants were determined by performing plaque assays on MDCK II cells by serial 10-fold dilutions in PBS containing 0.3% BSA.

### RT-PCR

Lung and liver were removed, and frozen immediately in liquid nitrogen. RNA was isolated from the organs using 1 ml of TriFast according to the protocol of the manufacturer (peQLab). The RNA was further purified by using RNeasy mini kit columns (Qiagen). One µg of each RNA preparation was reverse-transcribed using random-hexamer primers and reverse transcriptase. The reaction products were used to amplify the cDNAs by Taq polymerase for 30 cycles using the indicated primer pairs for mouse IFN-β (accession no. NM_010510, primers from positions 21–42 and 145–124), the mouse IFN-α family (accession no. NM_010504, primers from positions 46–68 and 557–534), mouse IFN-λ2 (accession no. NM_001024673, primers from positions 83–104 and 191–170), and mouse β-actin (accession no. X03672, primers from positions 1374–1396 and 1585–1564). RT-PCR products were separated by agarose electrophoresis, stained with ethidium bromide and visualized under UV light.

### Western blot analysis

Lung homogenates were prepared by grinding the tissue using a mortar and sterile quartz sand. Homogenates were lysed in buffer containing 50 mM Hepes (pH 7.3), 125 mM NaCl, 1% Nonidet P-40, 1 mM EDTA, 0.5% sodium deoxycholate, 0.1% SDS, 1 mM DTT, 100 units/ml of benzonase, and protease inhibitors as recommended by the manufacturer (Roche). Lysates were subjected to low speed centrifugation, and supernatants were diluted with concentrated gel loading buffer containing β-mercaptoethanol. Proteins were separated by SDS-polyacrylamide gel electrophoresis (10% gel) and transferred onto polyvinyliden-fluoride membranes (Millipore). The blots were probed with monoclonal mouse antibody specific for Mx1 [34] and monoclonal mouse antibody against actin (Sigma). Horseradish peroxidase-labeled secondary antibodies and the chemoluminescence detection system (Pierce) were used to detect primary antibodies. Signal quantification was done with a ChemiDoc XRS equipment (BioRad).

## References

[1] Yoneyama M, Fujita T (2007). Function of RIG-I-like receptors in antiviral innate immunity.. J Biol Chem.

[2] Takaoka A, Wang Z, Choi MK, Yanai H, Negishi H (2007). DAI (DLM-1/ZBP1) is a cytosolic DNA sensor and an activator of innate immune response.. Nature.

[3] Uematsu S, Akira S (2007). Toll-like receptors and Type I interferons.. J Biol Chem.

[4] Coccia EM, Severa M, Giacomini E, Monneron D, Remoli ME (2004). Viral infection and Toll-like receptor agonists induce a differential expression of type I and lambda interferons in human plasmacytoid and monocyte-derived dendritic cells.. Eur J Immunol.

[5] Pestka S, Krause CD, Walter MR (2004). Interferons, interferon-like cytokines, and their receptors.. Immunol Rev.

[6] van Pesch V, Lanaya H, Renauld JC, Michiels T (2004). Characterization of the murine alpha interferon gene family.. J Virol.

[7] Kotenko SV, Gallagher G, Baurin VV, Lewis-Antes A, Shen M (2003). IFN-lambdas mediate antiviral protection through a distinct class II cytokine receptor complex.. Nat Immunol.

[8] Sheppard P, Kindsvogel W, Xu W, Henderson K, Schlutsmeyer S (2003). IL-28, IL-29 and their class II cytokine receptor IL-28R.. Nat Immunol.

[9] Dumoutier L, Tounsi A, Michiels T, Sommereyns C, Kotenko SV (2004). Role of the interleukin (IL)-28 receptor tyrosine residues for antiviral and antiproliferative activity of IL-29/interferon-lambda 1: similarities with type I interferon signaling.. J Biol Chem.

[10] Zhou Z, Hamming OJ, Ank N, Paludan SR, Nielsen AL (2007). Type III interferon (IFN) induces a type I IFN-like response in a restricted subset of cells through signaling pathways involving both the Jak-STAT pathway and the mitogen-activated protein kinases.. J Virol.

[11] Brand S, Beigel F, Olszak T, Zitzmann K, Eichhorst ST (2005). IL-28A and IL-29 mediate antiproliferative and antiviral signals in intestinal epithelial cells and murine CMV infection increases colonic IL-28A expression.. Am J Physiol Gastrointest Liver Physiol.

[12] Lasfar A, Lewis-Antes A, Smirnov SV, Anantha S, Abushahba W (2006). Characterization of the mouse IFN-lambda ligand-receptor system: IFN-lambdas exhibit antitumor activity against B16 melanoma.. Cancer Res.

[13] Sommereyns C, Paul S, Staeheli P, Michiels T (2008). IFN-lambda (IFN-lambda) is expressed in a tissue-dependent fashion and primarily acts on epithelial cells in vivo.. PLoS Pathog.

[14] Ank N, Iversen MB, Bartholdy C, Staeheli P, Hartmann R (2008). An important role for type III Interferon (IFN-lambda/IL-28) in TLR-induced antiviral activity.. J Immunol.

[15] Muller U, Steinhoff U, Reis LF, Hemmi S, Pavlovic J (1994). Functional role of type I and type II interferons in antiviral defense.. Science.

[16] Staeheli P, Grob R, Meier E, Sutcliffe JG, Haller O (1988). Influenza virus-susceptible mice carry Mx genes with a large deletion or a nonsense mutation.. Mol Cell Biol.

[17] Tumpey TM, Szretter KJ, Van Hoeven N, Katz JM, Kochs G (2007). The Mx1 gene protects mice against the pandemic 1918 and highly lethal human H5N1 influenza viruses.. J Virol.

[18] Haller O, Frese M, Rost D, Nuttall PA, Kochs G (1995). Tick-borne thogoto virus infection in mice is inhibited by the orthomyxovirus resistance gene product Mx1.. J Virol.

[19] Koerner I, Kochs G, Kalinke U, Weiss S, Staeheli P (2007). Protective role of beta interferon in host defense against influenza A virus.. J Virol.

[20] Marie I, Durbin JE, Levy DE (1998). Differential viral induction of distinct interferon-alpha genes by positive feedback through interferon regulatory factor-7.. Embo J.

[21] Garcia-Sastre A, Egorov A, Matassov D, Brandt S, Levy DE (1998). Influenza A virus lacking the NS1 gene replicates in interferon-deficient systems.. Virology.

[22] Kochs G, Koerner I, Thiel L, Kothlow S, Kaspers B (2007). Properties of H7N7 influenza A virus strain SC35M lacking interferon antagonist NS1 in mice and chickens.. J Gen Virol.

[23] Hagmaier K, Jennings S, Buse J, Weber F, Kochs G (2003). Novel gene product of Thogoto virus segment 6 codes for an interferon antagonist.. J Virol.

[24] Ank N, West H, Paludan SR (2006). IFN-lambda: novel antiviral cytokines.. J Interferon Cytokine Res.

[25] Bouloy M, Janzen C, Vialat P, Khun H, Pavlovic J (2001). Genetic evidence for an interferon-antagonistic function of rift valley fever virus nonstructural protein NSs.. J Virol.

[26] Domanski P, Witte M, Kellum M, Rubinstein M, Hackett R (1995). Cloning and expression of a long form of the beta subunit of the interferon alpha beta receptor that is required for signaling.. J Biol Chem.

[27] Robek MD, Boyd BS, Chisari FV (2005). Lambda interferon inhibits hepatitis B and C virus replication.. J Virol.

[28] Decker T, Stockinger S, Karaghiosoff M, Muller M, Kovarik P (2002). IFNs and STATs in innate immunity to microorganisms.. J Clin Invest.

[29] Bartlett NW, Buttigieg K, Kotenko SV, Smith GL (2005). Murine interferon lambdas (type III interferons) exhibit potent antiviral activity in vivo in a poxvirus infection model.. J Gen Virol.

[30] Johnson TR, Mertz SE, Gitiban N, Hammond S, Legallo R (2005). Role for innate IFNs in determining respiratory syncytial virus immunopathology.. J Immunol.

[31] Horisberger MA, Staeheli P, Haller O (1983). Interferon induces a unique protein in mouse cells bearing a gene for resistance to influenza virus.. Proc Natl Acad Sci U S A.

[32] Chi B, Dickensheets HL, Spann KM, Alston MA, Luongo C (2006). Alpha and lambda interferon together mediate suppression of CD4 T cells induced by respiratory syncytial virus.. J Virol.

[33] Horisberger MA, de Staritzky K (1987). A recombinant human interferon-alpha B/D hybrid with a broad host-range.. J Gen Virol.

[34] Flohr F, Schneider-Schaulies S, Haller O, Kochs G (1999). The central interactive region of human MxA GTPase is involved in GTPase activation and interaction with viral target structures.. FEBS Lett.

